# Unilateral versus Bilateral T3 Ganglionectomy in Primary Palmar Hyperhidrosis Patients

**DOI:** 10.1055/a-2699-8163

**Published:** 2025-09-28

**Authors:** Beatrice Chia-Hui Shih, Duk Hwam Moon, Sungsoo Lee

**Affiliations:** 1 Department of Thoracic and Cardiovascular Surgery, Gangnam Severance Hospital, College of Medicine, Yonsei University, Gangnam-gu, Seoul, Korea

**Keywords:** hyperhidrosis, minimally invasive surgery (includes port access, minithoracotomy), practice guidelines, quality of life

## Abstract

**Background:**

Primary palmar hyperhidrosis (PPH) causes excessive hand sweating, impacting daily activities and quality of life. Endoscopic thoracic sympathectomy (ETS), including ganglionectomy, is a common treatment, but the risk of compensatory hyperhidrosis (CH) remains a concern. This study compares unilateral versus bilateral T3 ganglionectomy, focusing on differences in CH occurrence and patient satisfaction.

**Methods:**

We retrospectively analyzed 118 patients who underwent either unilateral or bilateral T3 ganglionectomy for PPH at our institution from November 2023 to January 2025. Data on patient characteristics and surgical outcomes were extracted from electronic medical records. Patient satisfaction and incidence of CH were assessed at postoperative 3 months.

**Results:**

Of the 118 patients with severe PPH, 77 underwent bilateral T3 ganglionectomy, and 41 received unilateral T3 ganglionectomy. No significant differences in baseline characteristics were observed between the groups. Postoperative satisfaction was higher in the unilateral group, with 93% reporting being “very satisfied” compared with 61% in the bilateral group ( *p* < 0.001). The unilateral group also had fewer incidences of CH, with 80% reporting no CH, while 43% of the bilateral group experienced mild CH ( *p* = 0.007). The most common areas affected by CH were the back, thighs, chest, abdomen, and hips. In the unilateral group, 7.5% showed improvement in contralateral sweating, with 22% necessitating contralateral ganglionectomy.

**Conclusion:**

This study is the first to compare the effectiveness and incidence of CH between unilateral and bilateral ETS for PPH. Our results show that 93% of unilateral ETS patients reported high satisfaction, compared with 61% in the bilateral group. Eighty percent of the unilateral group experienced no CH, while only 43% in the bilateral group reported mild CH. Statistically significant differences were observed in both satisfaction scores ( *p* < 0.001) and CH occurrence ( *p* = 0.007), suggesting unilateral ETS may provide better symptom relief with fewer adverse effects. Compared with prior studies, our cohort showed improved bilateral ETS outcomes, with only 48% developing CH. These findings indicate that unilateral ETS may be preferred for patients seeking higher satisfaction and reduced risk of CH, though further long-term studies are needed to confirm such results.

## Introduction

Primary palmar hyperhidrosis (PPH) is a condition characterized by excessive and uncontrollable sweating of the hands. It often emerges during puberty and persists into early adulthood. The constant dampness can lead to skin irritation, unpleasant odors, and difficulty performing routine tasks that require a dry grip. Beyond the physical discomfort, PPH can profoundly affect psychological well-being, causing heightened self-consciousness, social withdrawal, and anxiety in both personal and professional settings. This can dramatically impact academic, professional, and social performance. Over time, the cumulative physical and psychological burden of the condition can significantly diminish the quality of life. This underscores the need for effective management strategies.

Endoscopic thoracic sympathectomy (ETS), which includes ganglionectomy, sympathectomy, and sympathicotomy, treats PPH by altering its underlying pathology through targeted resection or interruption of the sympathetic chain at specific levels. In the absence of a universally accepted optimal surgical method or a specific level of interruption, the procedural choices are largely dependent on the surgeon's preference and/or experience. Furthermore, research has demonstrated that the incidence and severity of compensatory hyperhidrosis (CH)—a significant postoperative complication—vary depending on the specific ETS approach performed. 1 2 3

Although the precise mechanism of CH remains elusive, evidence suggests a heightened risk when the sympathetic trunk is interrupted at higher levels or when nerve pathways connected to the sympathetic ganglia are only partially blocked. 3 4 5 6 This incomplete interruption triggers compensatory sweat response in other body regions, diminishing the patient's quality of life and postoperative satisfaction. Despite numerous surgical modifications aimed at reducing CH, optimal prevention strategies remain inadequate.

Comparative studies of unilateral versus bilateral T3 ganglionectomy are scarce. In particular, the incidence of postoperative CH and the subsequent impact on the quality of life remain poorly defined for each approach. This study aims to make a comparative evaluation between unilateral and bilateral T3 ganglionectomy, focusing on the risk of postoperative CH and overall patient satisfaction.

## Methods

### Patients

We conducted a retrospective analysis of patients who underwent ETS, either unilateral or bilateral T3 ganglionectomy for PPH at a single institution from November 2023 to January 2025. Data on patient characteristics and surgical outcomes were extracted from electronic medical records. The primary outcomes of this study were postoperative satisfaction as well as the presence, severity, and location of CH. The study was approved by the local institutional review board at Gangnam Severance Hospital in South Korea, and informed consent was waived due to the study's retrospective design.

### Compensatory Hyperhidrosis and Satisfaction

CH was assessed 3 months postsurgery through a survey conducted in the outpatient clinic. The severity of CH was classified into the following categories: None, mild (not bothersome), moderate (sometimes bothersome), severe (often bothersome), or very severe (intolerable). Severity was assessed using the Hyperhidrosis Disease Severity Score (HDSS). Patient satisfaction was also measured at the 3-month postoperative period, with the following categories: Very satisfied, satisfied, moderately satisfied, dissatisfied, or very dissatisfied.

### Surgical Technique

All patients underwent ETS under general anesthesia, using either unilateral or bilateral simultaneous two-port video-assisted thoracoscopic surgery, with the side for unilateral procedures chosen by the patient. When a unilateral procedure was performed, the laterality was usually the patient's dominant hand, decided by patient discretion. The surgery involved making two 3-mm incisions on the operative side of the chest: One at the fourth intercostal space along the anterior axillary line for a 2-mm thoracoscope and another at the same level along the midaxillary line for surgical instruments. To deflate the lung, carbon dioxide was insufflated at a pressure of 5 mm Hg.

During the T3 ganglionectomy, the pleura was carefully incised between the third and fourth ribs. The white rami communicantes, arising from the third intercostal nerve, were meticulously followed to the sympathetic trunk. The T3 ganglion was then disconnected from the sympathetic trunk at both its rostral and caudal ends. The gray and white rami communicantes were cauterized at their entry and exit points into the ganglion. Antiadhesive material (hyaluronic acid, InterBlock, BioPlus Company, Seongnam, South Korea) was applied to the transected nerve sites. Then, a 10-Fr chest tube was inserted into the pleural cavity and removed after confirming complete air removal. Routine chest radiographs were performed in the operating room and again on postoperative day one to monitor for early signs of pneumothorax. Almost all patients were discharged on the same day or the following day.

### Statistical Analysis

Demographic and clinical data were expressed as mean ± standard deviation for continuous variables, or as frequency and percentage for categorical variables, as appropriate. Baseline characteristics between the two patient groups were compared using independent two-sample *t* -tests for continuous variables and chi-square tests for categorical variables. A *p* -value of less than 0.05 was considered statistically significant. All statistical analyses were performed using SAS version 9.4 (SAS Institute Inc., Cary, NC).

## Results

From November 2023 to January 2025, 118 patients with severe PPH were recruited. Seventy-seven patients received bilateral T3 ganglionectomy (the bilateral group) and 41 patients received unilateral T3 ganglionectomy (the unilateral group).

**Fig. 1:**
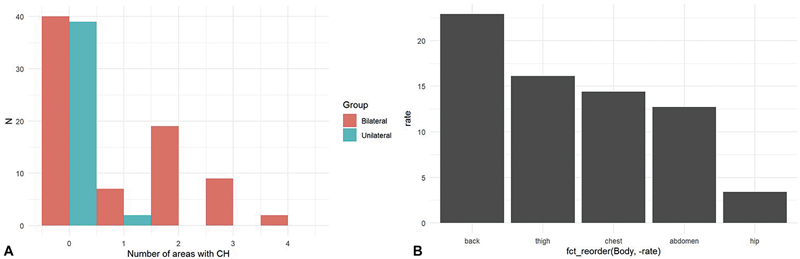
( **A** ) Number of areas affected by compensatory hyperhidrosis after bilateral versus unilateral T3 ganglionectomy. ( **B** ) Specific regions are affected in these patients.

There were no significant differences in age, sex, height, weight, or BMI between the two groups ( Table 1 ), indicating comparable physical metrics. However, postoperative satisfaction scores revealed notable differences. In the unilateral group, 93% of the patients reported being “very satisfied,” a striking contrast to 61% in the bilateral group ( Table 2 ). In the unilateral group, CH occurred less frequently, with 80% of patients reporting no signs of CH, 20% reporting mild signs, and confined to one body region. In contrast, CH was more prevalent in the bilateral group, with 43% of patients reporting mild symptoms (HDSS I), and a higher incidence of multiregion involvement ( Fig. 1A ). In both groups, the back, thighs, chest, abdomen, and hips were the most commonly affected areas ( Fig. 1B ). Statistically significant differences were observed between the two groups in satisfaction scores ( *p* < 0.001), number of affected areas ( *p* < 0.001), and CH occurrence ( *p* = 0.007). Additionally, in the unilateral group, further investigation of the contralateral hand revealed that 7.5% of patients experienced improved sweating while 93% observed no real change. Of the 93% of these patients, 22% subsequently underwent contralateral ganglionectomy, while 78% did not ( Table 3 ).

**Table 1:** Baseline clinical characteristics of bilateral versus unilateral T3 ganglionectomy patients

Characteristic	Bilateral ( *N* = 77 a )	Unilateral ( *N* = 41 a )	*p* -Value b
Age (years)	26 (8)	27 (9)	0.4
Sex			0.7
Female	40 (52%)	20 (49%)	–
Male	37 (48%)	21 (51%)	
Height (m)	1.68 (0.09)	1.67 (0.08)	0.7
Weight (kg)	65 (13)	66 (14)	> 0.9
BMI (kg/m ^2^ )	23.1 (3.2)	23.5 (4.0)	0.7

Abbreviation: BMI, body mass index.

a Mean (standard deviation); *n* (percentage).

b Wilcoxon rank-sum test; Pearson's chi-square test; Fisher's exact test.

**Table 2:** Compensatory hyperhidrosis and patient satisfaction in bilateral versus unilateral T3 ganglionectomy patients

Characteristic	Bilateral ( *N* = 77 a )	Unilateral ( *N* = 41 a )	*p* -Value b
Satisfaction score			< 0.001
Very satisfied	47 (61%)	38 (93%)	–
Satisfied	30 (39%)	1 (2.4%)	
Moderate	0 (0%)	2 (4.9%)	
Compensatory hyperhidrosis			0.007
Moderate (HDSS II)	4 (5.2%)	1 (2.4%)	–
Mild (HDSS I)	33 (43%)	7 (17%)	
None	40 (52%)	33 (80%)	
Number of CH regions			< 0.001
0	40 (52%)	33 (80%)	–
1	7 (9.1%)	8 (20%)	
2	19 (25%)	0 (0%)	
3	9 (12%)	0 (0%)	
4	2 (2.6%)	0 (0%)	

Abbreviations: CH, compensatory hyperhidrosis; HDSS, Hyperhidrosis Disease Severity Score.

a Mean (standard deviation); *n* (percentage).

b Wilcoxon rank-sum test; Pearson's chi-square test; Fisher's exact test.

**Table 3:** Progression of incidence of contralateral hyperhidrosis in unilateral T3 ganglionectomy patients

Characteristic	*N* = 41 a
Improved sweating in the contralateral hand	
Improved	3 (7.5%)
None	37 (93%)
Operation on the contralateral hand	
None	32 (78%)
Subsequent operation	9 (22%)

a *n* (percentage).

## Discussion

The present study demonstrated the clinical outcomes and patient follow-up after unilateral versus bilateral ETS at our institution. Through in-person surveys, we gathered data on patient satisfaction, the occurrence of CH, and its distribution. Using these data, we were able to compare the effectiveness and clinical outcomes of the two approaches.

To the best of our knowledge, this is the first study comparing the effectiveness and incidence of CH between unilateral and bilateral ETS. Our results reveal key differences in patient outcomes and overall satisfaction. Ninety-three percent of patients undergoing unilateral ETS reported being “very satisfied” compared with 61% undergoing bilateral ETS, suggesting that unilateral ganglionectomy may offer equal or superior symptom relief. Moreover, CH was less common in the unilateral group, with 80% experiencing no signs of CH and only 20% experiencing mild, one-region symptoms. In contrast, 43% of bilateral ETS patients reported mild CH, often affecting multiple regions. Statistically significant differences in satisfaction scores ( *p* < 0.001) and CH occurrence ( *p* = 0.007) underscore the impact of the surgical approach on patient outcomes. These findings indicate that unilateral ETS may limit the development of CH while maximizing patient satisfaction, which is an important consideration for both thoracic surgeons and patients when evaluating surgical options. Although the clear mechanism is not fully investigated, the positive effects concerning the contralateral hand in unilateral procedures in these patients may be due to anatomical variations and thoracic nerve connections to the other side of the body. Although the thoracic sympathetic ganglia and nerves control organs and tissues on the same side of the body, there are some connections between the sympathetic nervous systems of opposite sides of the body. These contralateral connections are seen in various cadaveric studies, so although these are not clearly visualized via thoracoscope, unilateral ganglionectomy seems to affect the opposite body's chains as well via crossing connections. 7

This study also revealed the occurrence of contralateral hyperhidrosis following unilateral ETS. Although 7.5% of patients noted improved sweating in the contralateral hand, 93% experienced no change—with 22% of these patients subsequently undergoing contralateral ganglionectomy. These findings suggest that additional surgery may be necessary for some patients to achieve optimal outcomes, raising questions about whether unilateral ETS alone is sufficient for long-term management of PPH. Adorisio et al reported in 2022 that in pediatric patients who underwent unilateral sympathectomy, only 12% showed CH and concluded that a single procedure on the dominant hand was effective enough to resolve bilateral problems. 8 Our study was in accordance with this previous study, but further research is needed to identify which patients might benefit from supplementary procedures.

Additionally, our study demonstrates that clinical outcomes for bilateral ETS in our cohort are notably improved compared with previous reports. Araújo et al (2009) observed that in a cohort of 80 patients who underwent bilateral ETS, 85% developed CH—classified as mild in 23 patients (33.8%), moderate in 23 patients (33.8%), and severe in 22 patients (32.4%). 9 Licht and Pilegaard reported in 2004 that in a cohort of 158 patients who underwent bilateral ETS, 89% developed CH. In contrast, only 48% of our bilateral ETS patients experienced any CH, with 43% showing only mild symptoms and 5.2% showing moderate symptoms. 10 This finding underlies the superior outcomes in our series.

One limitation of our study is its retrospective, cross-sectional design, which constrains the ability to track the progression of CH between unilateral and bilateral ETS over time. Additionally, patient satisfaction and CH occurrence were assessed using self-reported measures, which may introduce subjectivity, and thereby biases—patients might under- or overestimate their symptoms based on personal perceptions. Objective measurements of sweating could help address this limitation. We attempted to mitigate the risks associated with recall bias by reviewing medical records and promptly administering surveys during the 3-month postoperative outpatient follow-up.

Despite these limitations, our study offers valuable insights for both patients and surgeons, helping to identify those who may achieve reduced adverse outcomes and enhanced satisfaction. Our findings suggest that unilateral ETS is particularly promising for young patients with PPH—especially for those sensitive to CH and who prioritize higher postoperative satisfaction.

## Conclusion

This study shows that unilateral T3 ganglionectomy may be superior to bilateral ganglionectomy in patients with severe PPH. Patients who underwent unilateral ETS reported markedly higher satisfaction and experienced significantly lower incidence and severity of CH—a challenge in postoperative management for surgeons. Our results suggest that unilateral ETS may be sufficient for effective symptom control in many patients while minimizing CH. Also, only a small number of patients who received unilateral ETS required subsequent contralateral ETS, indicating that a staged approach may be appropriately done when needed. Therefore, unilateral ETS may be a more favorable strategy in the management of PPH, considering therapeutic benefit and postoperative satisfaction.
